# Personality Profiles Identify Depressive Symptoms over Ten Years? A Population-Based Study

**DOI:** 10.1155/2011/431314

**Published:** 2011-08-23

**Authors:** Kim Josefsson, Päivi Merjonen, Markus Jokela, Laura Pulkki-Råback, Liisa Keltikangas-Järvinen

**Affiliations:** ^1^IBS, Unit of Personality, Work, and Health Psychology, University of Helsinki, 00014 Helsinki, Finland; ^2^Finnish Institute of Occupational Health, 00250 Helsinki, Finland

## Abstract

Little is known about the relationship between temperament and character inventory (TCI) profiles and depressive symptoms. Personality profiles are useful, because personality traits may have different effects on depressive symptoms when combined with different combinations of other traits. Participants were from the population-based Young Finns study with repeated measurements in 1997, 2001, and 2007 (*n* = 1402 to 1902). TCI was administered in 1997 and mild depressive symptoms (modified Beck's depression inventory, BDI) were reported in 1997, 2001, and 2007. BDI-II was also administered in 2007. We found that high harm avoidance and low self-directedness related strongly to depressive symptoms. In addition, sensitive (NHR) and fanatical people (ScT) were especially vulnerable to depressive symptoms. high novelty seeking and reward dependence increased depressive symptoms when harm avoidance was high. These associations were very similar in cross-sectional and longitudinal analysis. Personality profiles help in understanding the complex associations between depressive symptoms and personality.

## 1. Introduction

The biosocial model of personality developed by Cloninger conceptualizes personality as the combination of two interrelated domains: temperament traits reflecting heritable and neurobiologically based differences in behavioral conditioning and character traits reflecting both neurobiological and sociocultural mechanisms of semantic and self-aware learning. Those domains are hypothesized to interact as a nonlinear dynamic system regulating the development of human psychological functions [1, 2].

According to Cloninger et al. [1, 3], temperament is related to heritable variation in automatic responses to environmental stimuli, especially to emotional ones, and is suggested to be involved in a specific neurotransmitter system of the brain. Temperament is characterized by novelty seeking (NS; a tendency toward exploratory activity and intense excitement in response to novel stimuli) that was originally hypothesized to be linked with low basal dopaminergic activity, harm avoidance (HA; a tendency to respond intensely to aversive stimuli and to avoid punishment and novelty) that was originally hypothesized to be linked with high serotonergic activity, reward dependence (RD; a tendency to respond intensely to reward and to learn to maintain rewarded behavior) that was originally hypothesized to be linked with low basal noradrenergic activity, and persistence (P) that has no special neural correlates [3]. However, Cloninger [1] has later acknowledged that the relationship between neurotransmitters and temperament is more complex than the originally postulated. 

The three character dimensions include self-directedness (SD), cooperativeness (CO) and self-transcendence (ST), and they reflect differences in higher cognitive functions underlying a person's self-concept, goals, and values [3]. SD describes the extent to which a person identifies the self as an autonomous individual. Typical people scoring high on SD are responsible, resourceful, and self-accepting [4]. People having low level of SD are blaming, aimless, and self-defeating. Cooperativeness expresses empathy and identification with other people and reflects the ability to cooperate with other people. Highly cooperative persons are tolerant, empathic, and helpful [4], while those scoring low on CO are prejudiced, insensitive, and hostile. Self-transcendence involves self-awareness of being an integral part of the unity of all things and is related to ones spirituality and universal values [3]. People having high level of ST are characterized as creative, intuitive, and spiritual [4], whereas a person scoring low on ST is typically conventional, analytical, and empirical. While temperament traits reflect stimulus-response characteristics underlying basic emotions, character depicts the maturity and coherent integration of the multiple facets of a person's personality in pursuit of particular goals and values in life. Together, they constitute personality as a dynamic and adaptive system with which individuals interpret and respond to their environment [3]. 

The extreme variants of the temperament traits of this dynamic system closely correspond to the traditional descriptions of different personality disorders, while immature character profile is used as a general marker of possible psychopathology [5]. This implies that the underlying structure of the normal adaptive personality traits is basically the same as that of the maladaptive personality traits [3, 6] and that the combinations and levels of traits make the difference between healthy and pathological personality. A combination of high HA and low SD has been convincingly associated with major depression in clinical populations [7–16]. HA has also been shown to modify the treatment effect of antidepressants on major depression [17]. Further, an association between high HA—low SD and depressive mood has been demonstrated in nonclinical samples, too [18–27]. Many of these studies have been based on general population samples [20, 23–27]. 

In general, it is important to know whether the findings derived from clinical samples can be generalized across healthy population. From the point of understanding the aspects of personality that predispose a person to depression, this is of high importance. TCI character profiles have been used in previous studies to explore the relationship between personality and well-being [28, 29]. However, to our knowledge, there is only one previous study that has used personality profiles to study the association between TCI and depression [30]. This study was cross-sectional, and there were 498 nonclinical participants who were all teachers. Personality profile in this study and in our study is defined as a combination of different personality traits within an individual. It is possible that, for example, the effect of high novelty seeking on an outcome measure is different in people who are low on harm avoidance than in people who are high on harm avoidance. Within individual personality profile is the only way to study this possibility. Gurpegui et al. [30] found that profiles with high harm avoidance or low self-directedness had higher frequency of depressive symptoms than other profiles. Similar results were observed with anxiety, social dysfunction, and somatic symptoms. 

Most of the before-mentioned studies are cross-sectional. There is no prospective, longitudinal population-based study to examine whether TCI personality profiles are associated with later depression. One challenge of cross-sectional studies is that temporary depressive mood might temporarily change personality and especially HA scores [27]. However, this is not necessarily true. For example, Cloninger et al. [23] found that all seven TCI-traits are more stable over one year interval than depressive mood. The greater stability of TCI compared to depression has also been reported by Richter et al. [31]. 

In this study we use temperament and character profiles, that is, a person-centered approach, in explaining the variation of depression. Examining personality profiles instead of single separate trait dimensions makes it possible to understand those processes within an individual that are associated with depression. This gives us more information than just examining differences between individuals using single traits. The present study was taken with a purpose to meet those challenges. We examine how temperament profiles as well as character profiles predict depressive symptoms cross-sectionally and prospectively four and ten years later in a population based cohort-study.

## 2. Methods

### 2.1. Participants

The Cardiovascular Risk in Young Finns Study started in 1980. The subjects for the original sample in 1980 (*N* = 3596) were selected randomly from six different age cohorts in the population register of the Social Insurance Institution, a database covering the whole population of Finland. The design of the study and the selection of the sample have been described in detail by Raitakari et al. [32]. The TCI-measurements for the present study were carried out in 1997. In 1997, the cohorts were 20, 23, 26, 29, 32 and 35 years old. Participants with missing information on any of the temperament and character traits were excluded. Some participants lacked these measures, because they did not fulfill the criteria of having answered a minimum of 50% of the items. Only 2% of the included participants had more than two missing items per one temperament or character trait. Depressive symptoms were measured in 1997, 2001, and 2007. Participants were excluded if they had not answered at least 50% of the depression items. At most, 0.3% of the included participants had more than two missing depression items. Statistical analyses on the relationship between temperament and character traits and depressive symptoms in different years were conducted independently of each other so the participants in each year formed highly overlapping but nonidentical groups.  Table 1 shows the frequency distribution of participants each year.

### 2.2. Measures

#### 2.2.1. Temperament and Character Inventory

We used version 9 of the TCI which has 240 items [33]. Instead of the original true/false response format, we used a 5 point Likert scale with response categories ranging from 1) absolutely false to 5) absolutely true. Temperament dimensions include harm avoidance (HA; 35 items, Cronbach's *α* = 0.92), novelty Seeking (NS; 40 items, *α* = 0.85), reward dependence (RD; 24 items, *α* = 0.80), and persistence (PS; 8 items, *α* = 0.64). Character dimensions include self-directedness (SD; 44 items, *α* = 0.89), cooperativeness (CO; 42 items, *α* = 0.91), and self-transcendence (ST; 33 items, *α* = 0.91).

#### 2.2.2. Tridimensional Temperament and Character Profiles

We followed the example of previous studies in forming the tridimensional personality profiles [2, 4, 30]. Temperament profiles consist of the eight possible combinations of high and low scores of novelty seeking, harm avoidance, and reward dependence. Character profiles consist of the eight possible combinations of high and low scores of self-directedness, cooperativeness, and self-transcendence. High and low scores were defined for all dimensions by median split. 

 As our aim was to capture the effects of extreme personality traits (high versus low), we decided to exclude participants with average temperament or character profile as was done in two previous studies [28, 29]. Average people form their own group, are usually flexible, and they do not demonstrate extreme characteristics [5]. Removing average people can be useful, because it reduces noise when studying the effect of extreme personality traits. A participant was labeled as average if he or she was in the middle third of the distribution for all three temperament traits or all three character traits. The final distribution of the profiles is shown in Table 1.

#### 2.2.3. Persistence

Originally, persistence was not included in the tridimensional temperament profiles [2, 4]. However, persistence has been found in previous studies to be associated with depressive symptoms [23, 25]. This is why we decided to analyze Persistence as an independent dimension.

#### 2.2.4. Mild Depressive Symptoms and Depressive Symptoms

Mild depressive symptoms were assessed using a modified version of Beck's depression inventory [34] in 1997, 2001, and 2007. In the original version of the BDI, subjects were asked to choose between one of four alternative descriptions of 21 items, with the descriptions of each item ranging from minimal to severe symptoms of depression. In the present study, the participants were asked to rate the second mildest descriptions of the original 21 items (e.g., “I often feel sad”) on a five-point scale ranging from totally disagree (1) to totally agree (5). For instance, an original BDI item could have the following four response options: (0) I do not feel sad, (1) I feel sad, (2) I am sad all the time and I cannot snap out of it, (3) I am so sad or unhappy that I cannot stand it. In our modified version we would select response option (1) and ask the participants to rate their agreement with it on a five-point Likert scale. Originally, these second mildest items were selected because they were expected to most accurately measure depressive symptoms among the normal population. Scale reliability was *α* = 0.91. 

In addition to mild depressive symptoms, in 2007 depressive symptoms were assessed using Beck's depression inventory-II (BDI-II). It measures self-reported depressive symptoms in adolescents and adults according to DSM-IV criteria for diagnosing depressive disorders [35]. Scale reliability in our data was *α* = 0.92. Each of the 21 items is rated on a four-point scale ranging from 0 to 3 and the total sum-score can range from 0 to 63. Scores from 0 to 13 represent “minimal” depression, scores from 14 to 19 are “mild”, scores from 20 to 28 are “moderate”, and scores from 29 to 63 are “severe” [35]. We also formed a dichotomous variable which grouped participants into those with at least mild depression (BDI-II) and those with minimal depression. This dichotomous depression variable was used in logistic regression analysis to evaluate the relative risk for depression in different temperament or character profiles.

Although BDI-II is a sum score, some participants with missing items were not removed. This was done because for a depressed person it is possible to be categorized as depressed with fewer than maximum number of items. Also, the percentage of participants with missing items was very small and the “answered at least 50% of the items”—criteria was in line with the criteria used with modified depressive symptoms scale assessing milder depressive symptoms.

### 2.3. Statistical Analyses

Analysis of variance (ANOVA) was used to examine differences between personality profiles. Sex and birth year were controlled when analyzing the profile differences. Possible profile × sex and profile × birth year interactions with depression scores were examined each year, but they were all nonsignificant in all the measurements. Profile comparisons were based on estimated marginal means, which were adjusted for sex and birth year. These adjustments were made because the original profiles were based on median scores unadjusted for sex and birth year. Bonferroni correction was used to correct for the multiple comparisons. We also used LSD-correction (equal to individual *t*-tests) when comparing different profiles. Persistence was studied using linear regression analysis and correlation coefficients. All analyses were conducted using SPPS for Windows version 18.

## 3. Results

### 3.1. Mild Depressive Symptoms (Modified BDI)

**Figure 1:**
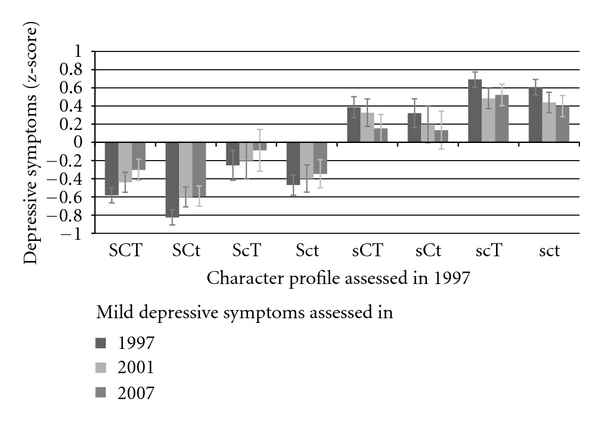
Standardized scores (mean = 0, SD = 1) of mild depressive symptoms (modified BDI) in different character combinations. 95% confidence intervals included. Sex and birth year were controlled. SCT = creative; SCt = organized; ScT = fanatical; Sct = autocratic; sCT = moody; sCt = dependent; scT = disorganized; sct = depressive.

Figure 1 shows the standardized mild depressive symptoms scores in 1997, 2001, and 2007 in the eight character profiles measured in 1997. Analysis of variance revealed highly significant differences between the profile groups in 1997 (*F* = 164.69, *P* < .001), 2001 (*F* = 51.85, *P* < .001), and 2007 (*F* = 40.03, *P* < .001). Bonferroni corrected comparison between groups showed that in all three measurement years the four profiles low on self-directedness (sct, scT, sCt, and sCT) had more frequently mild depressive symptoms than three profiles high in self-directedness (SCT, SCt, and Sct). The fanatical profile (ScT) was an exception; in all three measurement years fanatical people had more frequently mild depressive symptoms than organized (SCt) people. 

**Figure 2:**
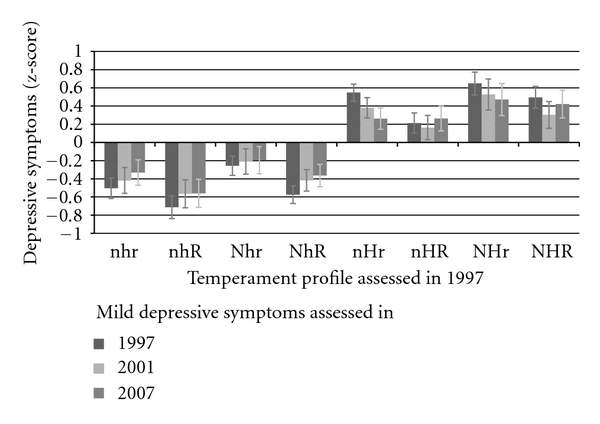
Standardized scores (mean = 0, SD = 1) of mild depressive symptoms (modified BDI) in different temperament combinations. 95% confidence intervals included. Sex and birth year were controlled. NHR = sensitive; NHr = explosive; NhR = passionate; Nhr = adventurous; nHR = cautious; nHr = methodical; nhR = reliable; nhr = independent.

Figure 2 shows the standardized mild depressive symptoms scores in 1997, 2001, and 2007 in the eight temperament profiles measured in 1997. Analysis of variance revealed highly significant differences between the profile groups in 1997 (*F* = 97.53, *P* < .001), 2001 (*F* = 35.39, *P* < .001), and 2007 (*F* = 29.41, *P* < .001). Bonferroni corrected comparison between groups showed that in all three measurement years the four profiles high on harm avoidance (nHR, nHr, NHR, and NHr) had more often mild depressive symptoms than the four profiles low on harm avoidance (nhr, nhR, Nhr, and NhR). Also, the adventurous profile (Nhr) exhibited more mild depressive symptoms in all three measurement years than reliable (nhR) profile.

### 3.2. Depressive Symptoms (BDI-II)

**Figure 3:**
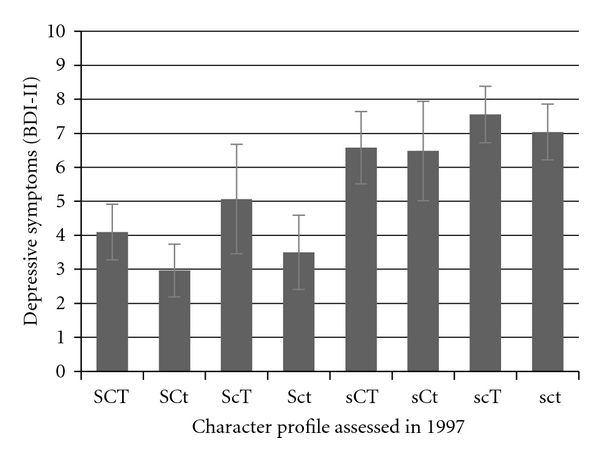
BDI-II depressive symptoms sum scores in different character combinations. Sex and birth year were controlled. SCT = creative; SCt = organized; ScT = fanatical; Sct = autocratic; sCT = moody; sCt = dependent; scT = disorganized; sct = depressive.

Figure 3 shows the depressive symptoms sum scores in year 2007 in the eight character profiles measured in 1997. Analysis of variance revealed highly significant differences between the profile groups (*F* = 15.41, *P* < .001). Bonferroni corrected comparison between groups showed that three profiles high on self-directedness (SCT, SCt, and Sct) had less frequently depressive symptoms than the three profiles low on Self-directedness (sct, scT, and sCT). Fanatical people (ScT) were again an exception; the fanatical profile did not differ significantly from any other character profile.

**Figure 4:**
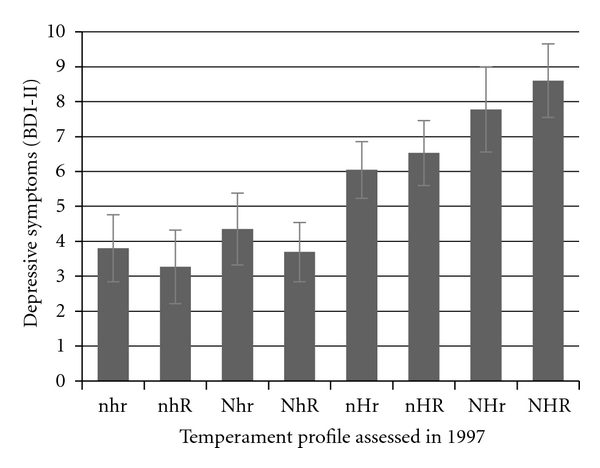
BDI-II depressive symptoms sum scores in different temperament combinations. Sex and birth year were controlled. NHR = sensitive; NHr = explosive; NhR = passionate; Nhr = adventurous; nHR = cautious; nHr = methodical; nhR = reliable; nhr = independent.

Figure 4 shows the depressive symptoms sum-scores in 2007 in the eight temperament profiles measured in 1997. Analysis of variance revealed highly significant differences between the profile groups (*F* = 15.16, *P* < .001). Bonferroni corrected comparison between groups showed that the four profiles high on harm avoidance (nHR, nHr, NHR, and NHr) had more frequently depressive symptoms than the three profiles low on harm avoidance (nhr, nhR, and NhR). In addition, the sensitive profile (NHR) had more frequently depressive symptoms than the methodical (nHr) profile.

### 3.3. Pairwise Comparison of Depressive Symptoms Scores in Different TCI-Profiles

Table 2 shows the pairwise profile comparisons for each TCI profile configuration for depressive symptoms. The comparisons show the effect of being high or low on a given trait when the other traits are held constant. The comparisons revealed the strong effect of harm avoidance and self-directedness on depressive symptoms. In all the comparisons people high on harm avoidance reported more frequently depressive symptoms than people low on harm avoidance. Also, in all the comparisons people high on self-directedness reported less frequently depressive symptoms than people low on Self-directedness. 

Other TCI-traits showed more mixed results. In most comparisons, people high on cooperativeness reported less frequently mild depressive symptoms (BDI_M) than people low on cooperativeness. However, cooperativeness did not have a significant effect on depressive symptoms (BDI-II) in 2007. Also, novelty seeking seemed to increase self-reported depressive symptoms. In all the comparisons people high on novelty seeking reported more frequently depressive symptoms than people low on novelty seeking. Not all the comparisons were significant but the trend was clear and consistent. Those having high novelty seeking reported more frequently high levels of depressive symptoms (BDI-II) especially when harm avoidance was high compared to those with low novelty seeking. Results were less clear for reward dependence. Those having high reward dependence reported less frequently higher levels of mild depressive symptoms (BDI_M) especially in 1997 and 2001 but in 2007 it did not have much significant effect. Also, Reward Dependence did not affect reported depressive symptoms (BDI-II). High self-transcendence consistently increased the probability of high reported depressive symptoms when both self-directedness and cooperativeness were high (SCT versus SCt). Mean difference in depressive symptoms between high and low self-transcendence was also consistently rather large when only self-directedness was high (ScT versus Sct) but due to the small *N* in the profile groups, the mean difference was not significant in three of the four measurements.

### 3.4. TCI-Profiles in 1997 Predicting BDI-II Depression in 2007

Table 3 shows the frequency of depression (BDI-II) in personality profiles in 2007. “No depression” means that a person's depressive symptoms score is at most 13. “Depressed” means that a person's depressive symptoms score is at least 14. The percentage of depressed people is higher (All %) in all those profiles where harm avoidance is high than in those where harm avoidance is low. Interestingly, in addition to harm avoidance, reward dependence, and novelty seeking seem to contribute to the frequency of depression; sensitive people (NHR) are more frequently depressed (All %) than methodical (nHr), explosive (NHr), or cautious (nHR) people. According to the odds ratios, methodical people (nHr) are not significantly more frequently depressed than reliable (nhR) people. Sensitive people (NHR) have over 5-times higher odds of being depressed and also explosive (NHr) and cautious (nHR) people have over 3 times greater odds to be depressed than reliable (nhR) people. The number of men in certain profiles is not large but still the difference between the most frequently depressed profile (NHR, 45.5%) and least frequently depressed profile (nhR, 3.9%) in men is very large in terms of depression frequency. Both in men and women sensitive (NHR) people have the highest frequency of depression. Cautious women (nHR) are rather often depressed (19.8%) but this is not true for cautious men (7.1%).

Also the character profiles show differences in depression frequency. Except for the fanatical (ScT) profile, people high on self-directedness (SCT, SCt, and Sct) belonged less frequently in depressed group than people low on self-directedness (sct, scT, sCt, and sCT). If self-directedness and Cooperativeness are held constant (e.g., SCT versus SCt in Table 3) in all the contrasts the profile higher on self-transcendence is more frequently depressed (All %). Fanatical men and women (ScT) were more frequently depressed than other profiles high on Self-directedness, and, in men, fanatical profile was most often depressed (19.0%). According to percentages, disorganized (scT) or depressive (sct) women were more frequently depressed than disorganized or depressive men, respectively. According to the odds ratios, fanatical people (ScT) and those low on self-directedness (sCT, sCt, scT, and sct) were more often depressed than organized (SCt) people. Disorganized people (scT) were the most frequently depressed group according to the odds ratios.

### 3.5. The Relationship between Depressive Symptoms and Persistence

The linear relationship between Persistence and depressive symptoms was explored using correlation coefficients and linear regression. Correlations between Persistence and mild depressive symptoms in 1997, 2001, and 2007 were −.07, −.01, and  .00, respectively. Correlation between persistence and depressive symptoms (BDI-II) in 2007 was  .02. Only the correlation with mild depressive symptoms in 1997 was significant at  .05 level.

Table 4 shows the results of linear regression analysis for persistence predicting depressive symptoms. The association between persistence and depressive symptoms was negative in 1997 and positive in 2001 and 2007. Three of the seven regression coefficients for persistence were statistically significant. Persistence explained, at best, 0.4% of the variation in depressive symptoms.

## 4. Discussion

The most important findings of this study were the effect of novelty seeking and reward dependence temperament traits on depressive symptoms in addition to harm avoidance, and the increased probability for BDI-II depression (Table 3) of the fanatical character profile (ScT) despite having high self-directedness. Sensitive people (NHR) had more frequently depressive symptoms (BDI-II) than methodical people (nHr) although both had high harm avoidance. In addition, the current results confirmed the findings of previous studies about the strong impact of high harm avoidance and low self-directedness on the frequency of depressive symptoms (e.g., [7–10, 23]). High level of depressive symptoms could be predicted with high harm avoidance and low self-directedness strongly and consistently both cross-sectionally and over time. Our results also confirmed the findings of previous studies according to which persistence was positively associated with depressive symptoms when baseline depressive symptoms are controlled [23, 25].

The use of personality profiles led to an important finding: the effect of harm avoidance and self-directedness on depressive symptoms depends on the configuration of the other temperament and character traits. It is interesting to contrast our results with those of Gurpegui et al. [30] who also used TCI personality profiles in their nonclinical psychopathology study although they used the short version of TCI (TCI-125) and a true/false response format which reduces variance compared to a five-point Likert-scale. The differences found by them in depressive symptoms scores between personality profiles were mostly due to harm avoidance and self-directedness. People with sensitive (NHR), explosive (NHr), or methodical (nHr) temperament profile had more frequently depressive symptoms than others. Also, people with moody (sCT), dependent (sCt), disorganized (scT), or depressive (sct) character profiles had more frequently depressive symptoms than others. Other TCI-traits besides HA and SD did not have a consistent significant effect on depressive symptoms. Our results are different in this aspect, because we found that all seven TCI-traits had at least some effect on the frequency of depressive symptoms between different profiles. 

From the temperament profiles sensitive (NHR) temperament was the best predictor of BDI-II depression 10-years later, increasing the risk to almost 6-fold. Also having explosive (NHr) or cautious (nHR) temperament profile increased the risk of BDI-II depression to over 3-fold. Regarding the character traits, disorganized (scT) individuals had over 5-times greater risk to become depressed compared to organized (SCt) persons. Also, moody (sCT), depressive (sct), fanatical (ScT) or dependent (sCt) character profiles predicted over threefold risk of later BDI-II depression. Thus those having disorganized (scT) character and sensitive (NHR) temperament profile might be most vulnerable for future depression. Also, fanatical people (ScT) had an increased risk for BDI-II depression even though they were high on self-directedness. Fanatical people can be characterized as independent and paranoid, and being projective of blame [36]. 

Novelty seeking and reward dependence, in turn, did not have a consistent effect on BDI-II depression in 2007 when harm avoidance was low. However, when harm avoidance was high, both high novelty seeking and high reward dependence increased the probability for having BDI-II depression. Sensitive people (NHR) were most likely to be depressed according to BDI-II. Sensitive people respond intensely to aversive (HA) and novel (NS) stimuli, and to social reward and punishment (RD). This combination seems to make them especially vulnerable to depression. 

Temperament traits, especially harm avoidance, might be related to emotional vulnerability to depression, whereas character traits, especially self-directedness, might be associated with executive cognitive functions that protect a person from depression [23]. However, high harm avoidance is associated with a wide range of psychopathology and it is not typical only of depression [30]. All in all, it seems that individuals with depression are likely to be both anxiety-prone (i.e., high in harm avoidance) and immature (i.e., low in self-directedness). Maturity refers to the character configuration typical of healthy middle-aged individuals, which is characterized by high Self-directedness and high Cooperativeness [2, 3, 28, 29]. It is consistent with what is described as healthy or health-promoting personality traits, as proposed for DSM-V [37].

Cooperativeness, self-transcendence, reward dependence, and novelty seeking also had an impact on depressive symptoms in addition to harm avoidance and self-directedness. Cooperativeness was negatively associated with mild depressive symptoms cross-sectionally and over four and ten years. However, cooperativeness was not significantly associated with BDI-II depressive symptoms over ten years. This is in line with previous research which has found that cooperativeness is cross-sectionally associated with depression but does not predict later depression [23]. However, our results show that cooperativeness is negatively associated with mild depressive symptoms over time but not with more severe self-reported depressive symptoms. 

Using personality profiles proved to be useful in examining the effect of Self-transcendence on depressive symptoms. When self-directedness was low, self-transcendence, by itself, did not have a significant effect on depressive symptoms. However, when self-directedness was high, Self-transcendence was positively associated with the mean levels of depressive symptoms. This might explain why some earlier studies have found a positive association between Self-transcendence and depression [7, 10, 14] and some have not found an association [11, 27].

The previous studies regarding the role of novelty seeking or reward dependence as a predictor of depression are contradictory. Some studies have found that novelty seeking is negatively associated with depression [7, 15, 26, 31] while some studies have reported a positive association [14, 19]. Similarly, in some studies reward dependence has been found to be negatively associated with depression [14, 21] but not in all [11]. Our results suggest that the association between novelty seeking and depressive symptoms is positive but the magnitude depends on the personality profile. High novelty seeking was a significant predictor of high levels of BDI-depressive symptoms (Table 2) only when harm avoidance was high. As regards to reward dependence, our results suggest that it is negatively associated with mild depressive symptoms but not significantly with BDI-II depressive symptoms, thus giving support to the previous findings. 

Another key finding of our study was that the association between temperament and character traits and depressive symptoms might depend on the definition of depressive symptoms themselves. For example, when mild depressive symptoms were used as a depressive symptoms measure, the effect of novelty seeking was quite similar in all personality profiles. However, when BDI-II depressive symptoms were used as a depressive symptoms measure, novelty seeking was significantly associated with depressive symptoms only in the profiles with high harm avoidance. Furthermore, reward dependence was negatively associated with mild depressive symptoms but positively associated with BDI-II depressive symptoms when harm avoidance was high. In addition, cooperativeness was consistently positively associated with mild depressive symptoms but not with BDI-II depressive symptoms. 

 The temperament and character profiles were associated with depressive symptoms cross-sectionally and also four or ten years later. This is an important finding since it implies that cross-sectional analyses focusing on the association between personality and depressive symptoms give valuable information and predictions can be made using them. TCI profiles identified depressive symptoms both cross-sectionally and prospectively. However, it is not clear what the clinical significance of this finding is. A replication of this study is needed using clinically verified depression as an outcome instead of depressive symptoms. 

Our results are in agreement with neurobiological findings according to which a personality trait might not be related to a single neurotransmitter system [38]. Modulation and interaction are very common in brain functions and the effects of neurotransmitters on behavior are not linear [38]. This is exactly what our results suggest; the effects of different temperament and character traits are not strictly linear or independent of each other but depend on the combination and levels of other traits. Our results suggest that the strong effects of harm avoidance and self-directedness on depressive symptoms are very dominating and can mask the effects of other temperament and character traits if the interactions between the traits are not taken into account. When these interactions are taken into account, the complex relationship between personality and depressive symptoms is better understood, as we have shown. In future studies and with a sufficiently large number of participants, it would be useful to study the combination of harm avoidance and the maturity of personality because mature personality forms a preventive shield protecting oneself of developing mental disorders [3, 37].

Our study was not without limitations. Cloninger's theory sees personality as an adaptive system where the temperament traits interact, and where the outcomes of temperament are modified by the maturity levels of character traits. Temperament and character are not independent of each other, implying that when we assess temperament we also assess character to some extent. Therefore, our temperament and character profiles do not represent pure temperament or character but a combination of both. It would be extremely interesting in future studies to explore the combined temperament × character profiles. This, however, leads to 8 × 8 = 64 different profiles which means that a large number of participants is needed to avoid profiles with zero or only a few participants. The associations between temperament traits and depression risk may also depend on social and environmental circumstances [39], and the association between character and well-being might be influenced by culture [29]. This context-specificity implies that the associations between personality and depression might be, at least partly, culture specific and not fully generalizable from one country to another.

Given the longitudinal design, some associations might have been affected by selective study attrition. We tried to lower the probability for this possibility by not requiring all the participants to have full data in all the measurement years which makes the study sample less selective. In addition, both personality and depressive symptoms were self-rated. It is possible that personality affects a person's depressive symptoms rating or vice versa. The clinical significance of our results is questionable, since it cannot be said how many of the participants would fill the criteria for a clinical depression diagnosis. It is also questionable if causal attributions can be inferred from our study, since we did not control for baseline depressive symptoms. Nevertheless, our study gives a rather comprehensive picture of the association between personality profiles and depressive symptoms. Our aim was not to predict depressive symptoms per se but to identify the differences between TCI profiles in the frequency of depressive symptoms.

## 5. Conclusions

In summary, we have shown the importance of personality profiles in studying the vulnerability to depressive symptoms cross-sectionally and over time. We showed that in addition to disorganized (scT) character profile carriers, also those having moody (sCT), depressive (sct), fanatical (ScT), or dependent (sCt) character profiles are vulnerable to developing BDI-II depression. Especially the fanatical profile is interesting since high self-directedness usually protects a person from depression. From temperament traits it seems that it is not high harm avoidance alone, rather it is high harm avoidance combined with other high temperament traits that increases frequency of depressive symptoms. The reason for this is not clear but it might refer to inner conflicts in the person's motivational systems, that is, a combination of anxiousness and a preference for novelty and social rewards. Those having sensitive (NHR), explosive (NHr) or cautious (nHR) temperament profiles are in increased danger to have BDI-II depression. Interestingly, the methodical (nHr) profile has only slightly increased risk for BDI-II depression although their harm avoidance is high. Our results highlight the importance of the interaction of harm avoidance and self-directedness with the other TCI-traits when assessing the risk for depressive symptoms.

## Figures and Tables

**Table 1:** Frequency distribution of TCI profiles.

	*N* 1997 (women/men)	*N* 2001 (women/men)	*N* 2007, BDI_M (women/men)	*N* 2007, BDI-II (women/men)
*Temperament*				
NHR—sensitive	210 (186/24)	166 (152/14)	158 (147/11)	158 (147/11)
NHr—explosive	177 (92/85)	112 (64/48)	107 (59/48)	107 (59/48)
NhR—passionate	310 (226/84)	245 (186/59)	231 (172/59)	231 (172/59)
Nhr—adventurous	249 (100/149)	166 (73/93)	149 (74/75)	149 (74/75)
nHR—cautious	240 (197/43)	193 (159/34)	200 (172/28)	200 (172/28)
nHr—methodical	316 (155/161)	258 (128/130)	241 (121/120)	239 (121/118)
nhR—reliable	180 (108/72)	139 (88/51)	144 (93/51)	144 (93/51)
nhr—independent	220 (74/146)	163 (56/107)	174 (62/112)	174 (62/112)
*Character*				
SCT—creative	336 (251/85)	268 (211/57)	254 (202/52)	253 (202/51)
SCt—organized	344 (192/152)	255 (149/106)	265 (157/108)	265 (157/108)
ScT—fanatical	87 (52/35)	75 (48/27)	61 (40/21)	61 (40/21)
Sct—autocratic	189 (72/117)	137 (52/85)	134 (55/79)	134 (55/79)
sCT—moody	181 (147/34)	141 (122/19)	147 (122/25)	147 (122/25)
sCt—dependent	94 (64/30)	76 (49/27)	75 (55/20)	75 (55/20)
scT—disorganized	346 (210/136)	248 (167/81)	233 (155/78)	232 (155/77)
sct—depressive	325 (150/175)	242 (108/134)	235 (114/121)	235 (114/121)

Total	1902 (1138/764)	1442 (906/536)	1404 (900/504)	1402 (900/502)

In 1997 and 2001, depressive symptoms were assessed by the modified version of the BDI only (see methods for details).

In 2007 depressive symptoms were assessed by both the original BDI-II and modified BDI NHR = sensitive; NHr = explosive; NhR = passionate; Nhr = adventurous; nHR = cautious; nHr = methodical; nhR = reliable; nhr = independent.

SCT = creative; SCt = organized; ScT = fanatical; Sct = autocratic; sCT = moody; sCt = dependent; scT = disorganized; sct = depressive.

**Table 2:** Pairwise comparison of depressive symptom scores between groups of various temperament and character profiles.

	BDI_M 1997		BDI_M 2001		BDI_M 2007		BDI-II 2007	
	MD	*P*	MD	*P*	MD	*P*	MD	*P*
*Novelty Seeking*								
NHR versus nHR	.283	.000	.140	.152	.159	.110	2.074	.002
NHr versus nHr	.101	.207	.146	.163	.211	.053	1.728	.021
NhR versus nhR	.137	.087	.149	.129	.196	.048	.424	.534
Nhr versus nhr	.247	.002	.209	.040	.136	.000	.549	.444
*Harm Avoidance*								
NHR versus NhR	1.072	.000	.719	.000	.786	.000	4.912	.000
NHr versus Nhr	.905	.000	.737	.000	.667	.000	3.420	.000
nHR versus nhR	.926	.000	.728	.000	.823	.000	3.262	.000
nHr versus nhr	1.051	.000	.800	.000	.593	.000	2.241	.000
*Reward Dependence*								
NHR versus NHr	−.152	.084	−.225	.048	−.050	.672	.831	.307
NhR versus Nhr	−.319	.000	−.207	.028	−.169	.088	−.660	.331
nHR versus nHr	−.334	.000	−.219	.014	.001	.993	.485	.441
nhR versus nhr	−.210	.015	−.147	.172	−.229	.032	−.536	.465
*Self-directedness*								
SCT versus sCT	−.968	.000	−.766	.000	−.456	.000	−2.480	.000
SCt versus sCt	−1.146	.000	−.798	.000	−.725	.000	−3.515	.000
ScT versus scT	−.944	.000	−.684	.000	−.608	.000	−2.487	.007
Sct versus sct	−1.076	.000	−.837	.000	−.744	.000	−3.537	.000
*Cooperativeness*								
SCT versus ScT	−.328	.001	−.239	.040	−.215	.100	−.967	.290
SCt versus Sct	−.355	.000	−.203	.033	−.245	.012	−.535	.433
sCT versus scT	−.305	.000	−.157	.096	−.367	.000	−.975	.150
sCt versus sct	−.286	.002	−.242	.040	−.264	.031	−.558	.515
Self-transcendence								
SCT versus SCt	.244	.000	.162	.040	.289	.000	1.133	.046
ScT versus Sct	.217	.033	.197	.125	.259	.068	1.564	.116
sCT versus sCt	.066	.510	.129	.311	.020	.875	.098	.915
scT versus sct	.085	.162	.044	.588	.123	.149	.514	.388

BDI_M = modified Beck's depression index; BDI = original Beck's depression index Comparisons based on LSD-adjusted marginal means in ANOVA.

Results are adjusted for sex and cohort.

NHR = sensitive; NHr = explosive; NhR = passionate; Nhr = adventurous; nHR = cautious; nHr = methodical; nhR = reliable; nhr = independent.

SCT = creative; SCt = organized; ScT = fanatical; Sct = autocratic; sCT = moody; sCt = dependent; scT = disorganized; sct = depressive.

**Table 3:** Results of logistic regression where temperament or character profile was the independent variable and binary BDI-II depression score (not depressed = 0 and >13 = 1) the dependent variable.

	All %	Women %	Men %	Odds ratio (All)	CI (All)	*P* (All)	Odds ratio (women)	CI (women)	*P* (women)	Odds ratio (men)	CI (men)	*P* (men)
*Temperament*												
NHR—sensitive	25.9	24.5	45.5	5.78	2.58–12.95	.000	4.74	1.90–11.78	.001	20.01	3.06–130.92	.002
NHr—explosive	17.8	16.9	18.8	3.89	1.63–9.31	.002	3.09	1.06–9.04	.040	6.71	1.33–33.75	.021
NhR—passionate	6.1	5.8	6.8	1.06	.43–2.59	.907	.89	.31–2.53	.822	1.60	.27–9.28	.603
Nhr—adventurous	6.7	8.1	5.3	1.24	.47–3.26	.658	1.31	.40–4.24	.657	1.36	.24–7.81	.733
nHR—cautious	18.0	19.8	7.1	3.65	1.63–8.17	.002	3.62	1.46–9.01	.006	1.98	.26–15.22	.511
nHr—methodical	11.3	12.4	10.2	2.23	.98–5.07	.057	2.11	.78–5.69	.139	2.54	.54–12.04	.240
nhR—reliable	5.6	6.5	3.9	reference			reference			reference		
nhr—independent	6.3	8.1	5.4	1.21	.47–3.12	.694	1.29	.37–4.43	.691	1.32	.25–6.91	.743
*Character*												
SCT—creative	7.5	8.4	3.9	1.58	.75–3.34	.231	1.73	.72–4.12	.218	1.00	.17–5.70	.997
SCt—organized	4.5	5.1	3.7	reference			reference			reference		
ScT—fanatical	14.8	12.5	19.0	3.59	1.43–8.99	.006	2.72	.84–8.86	.096	6.48	1.44–29.16	.015
Sct—autocratic	4.5	5.5	3.8	1.06	.39–2.90	.914	1.08	.28–4.26	.908	.93	.20–4.31	.924
sCT—moody	18.4	18.9	16.0	4.43	2.15–9.11	.000	4.44	1.90–10.38	.001	4.65	1.06–20.44	.042
sCt—dependent	14.7	16.4	10.0	3.46	1.45–8.25	.005	3.65	1.33–10.05	.012	2.78	.47–16.56	.263
scT—disorganized	20.7	23.9	14.3	5.56	2.86–10.81	.000	6.16	2.75–13.80	.000	4.72	1.43–15.56	.011
sct—depressive	14.5	17.5	11.6	3.83	1.92–7.62	.000	4.01	1.69–9.50	.002	3.56	1.12–11.31	.031

Depression measured by Beck's original depression index (BDI-II).

Odds ratio and *P* value based on binary logistic regression where depression (0 or 1) was the the outcome and personality profile the predictor. Odds ratios based on combined sample of men and women cohort and sex were controlled in the regression analysis.

Birth year was not controlled when calculating the percentages.

**Table 4:** Regression coefficients of persistence predicting depressive symptoms.

	BDI_M 1997			BDI_M 2001			BDI_M 2007			BDI-II 2007		
	*B* (SE)	*P*	Δ*R* ^2^	*B* (SE)	*P*	Δ*R* ^2^	*B* (SE)	*P*	Δ*R* ^2^	*B* (SE)	*P*	Δ*R* ^2^
Step 1												
Persistence	−.11 (.04)	.010	.004	−.01 (.05)	.881	.000	.01 (.05)	.766	.000	.36 (.32)	.261	.001
Step 2												
Persistence				.07 (.04)	.042	.001	.07 (.04)	.065	.002	.66 (.29)	.023	.003

Δ*R* ^2^ = change in *R* ^2^ compared to the model with only control variables.

Step 1 = effect of persistence when sex and birth year were controlled. Step 2 = effect of persistence when sex, birth year, and mild depressive symptoms in 1997 were controlled.

BDI_M = mild depressive symptoms (see Section 2).

BDI-II = depressive symptoms measured by BDI-II.
